# Radiomic and Dosiomic Features for the Prediction of Radiation Pneumonitis Across Esophageal Cancer and Lung Cancer

**DOI:** 10.3389/fonc.2022.768152

**Published:** 2022-02-16

**Authors:** Chanon Puttanawarut, Nat Sirirutbunkajorn, Narisara Tawong, Chuleeporn Jiarpinitnun, Suphalak Khachonkham, Poompis Pattaranutaporn, Yodchanan Wongsawat

**Affiliations:** ^1^Chakri Naruebodindra Medical Institute, Faculty of Medicine Ramathibodi Hospital, Mahidol University, Samut Prakan, Thailand; ^2^Brain-Computer Interface Laboratory, Department of Biomedical Engineering, Faculty of Engineering, Mahidol University, Nakhorn Pathom, Thailand; ^3^Department of Diagnostic and Therapeutic Radiology, Faculty of Medicine, Ramathibodi Hospital, Mahidol University, Bangkok, Thailand

**Keywords:** radiotherapy, dosiomic, radiomic, machine learning, DVH, radiation pneumonitis, esophageal cancer

## Abstract

**Purpose:**

The aim was to investigate the advantages of dosiomic and radiomic features over traditional dose-volume histogram (DVH) features for predicting the development of radiation pneumonitis (RP), to validate the generalizability of dosiomic and radiomic features by using features selected from an esophageal cancer dataset and to use these features with a lung cancer dataset.

**Materials and Methods:**

A dataset containing 101 patients with esophageal cancer and 93 patients with lung cancer was included in this study. DVH and dosiomic features were extracted from 3D dose distributions. Radiomic features were extracted from pretreatment CT images. Feature selection was performed using only the esophageal cancer dataset. Four predictive models for RP (DVH, dosiomic, radiomic and dosiomic + radiomic models) were compared on the esophageal cancer dataset. We further used a lung cancer dataset for the external validation of the selected dosiomic and radiomic features from the esophageal cancer dataset. The performance of the predictive models was evaluated by the area under the curve (AUC) of the receiver operating characteristic curve (ROCAUC) and the AUC of the precision recall curve (PRAUC) metrics.

**Result:**

The ROCAUCs and PRAUCs of the DVH, dosiomic, radiomic and dosiomic + radiomic models on esophageal cancer dataset were 0.67 ± 0.11 and 0.75 ± 0.10, 0.71 ± 0.10 and 0.77 ± 0.09, 0.71 ± 0.11 and 0.79 ± 0.09, and 0.75 ± 0.10 and 0.81 ± 0.09, respectively. The predictive performance of the dosiomic- and radiomic-based models was significantly higher than that of the DVH-based model with respect to esophageal cancer. The ROCAUCs and PRAUCs of the DVH, dosiomic, radiomic and dosiomic + radiomic models on the lung cancer dataset were 0.64 ± 0.18 and 0.37 ± 0.20, 0.67 ± 0.17 and 0.37 ± 0.20, 0.67 ± 0.16 and 0.45 ± 0.23, and 0.68 ± 0.16 and 0.44 ± 0.22, respectively. On the lung cancer dataset, the predictive performance of the radiomic and dosiomic + radiomic models was significantly higher than that of the DVH-based model. However, the PRAUC of the dosiomic-based model showed no significant difference relative to the corresponding RP prediction performance on the lung cancer dataset.

**Conclusion:**

The results suggested that dosiomic and CT radiomic features could improve RP prediction in thoracic radiotherapy. Dosiomic and radiomic feature knowledge might be transferrable from esophageal cancer to lung cancer.

## Introduction

In thoracic radiation therapy, organs at risk, such as the lungs, are the limiting factors of radiation treatment due to radiation toxicity. Radiation pneumonitis (RP) is one type of lung toxicity. Many studies have tried to develop RP prediction models based on dose volume histograms (DVHs) and/or the clinical profiles of patients (1–3). However, DVHs and clinical factors are only some of the many pieces of information that can be extracted from patients.

Recently, quantitative image features such as the dosiomic (quantitative features of dose distribution) and/or radiomic features of computed tomography (CT) images have been reported to improve the performance of prediction models for radiation toxicity (4–8). Dosiomic features contain more dose distribution information than DVH features and have been shown to be able to improve toxicity prediction in radiation therapy. Information that can be used for the prediction of RP can also be found in CT images. For example, interstitial lung disease was found to be a risk factor for RP (9–11). RP prediction models for lung cancer have also been shown to benefit from the use of radiomic features obtained from CT images (6–8). The quantitative imaging features of fluorine 18 fluorodeoxyglucose (FDG) positron emission tomography (PET)/CT were previously studied in esophageal cancer patients (12). While the radiomic features from CT were not found to be significant, the radiomic features from FDG-PET SUV were significantly associated with grade 2 RP. However, only a subset of radiomics features in CT images was explored.

Studies of dosiomic and radiomic features can result in feature selection bias, as demonstrated by a systematic review by Chalkidou et al., who generated 100 random features and found that 10% of the features were significant predictors (13). Furthermore, some random variables achieved higher performance metric scores than other significant features, as reported in other studies. To reduce the false-positive rates in radiomic studies, external validation was recommended (14–18).

This study aimed to investigate the benefit of using radiomic and dosiomic features in an RP prediction model for esophageal cancer patients. We compared four predictive models with DVH features, dosiomic features, radiomic features and combined dosiomic and radiomic features. Furthermore, to investigate the generalizability of dosiomic and radiomic features, we incorporated an external dataset with lung cancer patients and investigated a predictive model using features selected from esophageal cancer data.

## Material and Methods

### Data

The CT images, ROIs, and 3D dose distributions of 333 esophageal cancer patients and 110 lung cancer patients >15 years of age who were treated with radiation therapy from 2011 to 2019 were extracted from the Varian Eclipse v16.1 treatment planning system (TPS) (Varian Medical Systems, Palo Alto, CA) at the Ramathibodi Hospital at Mahidol University. The study was approved by the ethical committee of the Ramathibodi Hospital at Mahidol University (IRB MURA2021/283). Patients with previous histories of thoracic radiation therapy, diagnoses of interstitial lung disease, follow-up times under one year, no treatment data or diagnoses of lung metastasis within one year were excluded from the study. After exclusion, 101 patients and 93 patients had esophageal cancer and lung cancer, respectively. The clinical and treatment characteristics are shown in **Table 1**. All dose distribution were calculated by Anisotropic Analytical Algorithm (AAA) from Varian Eclipse TPS. The script for the extraction of the treatment plan from the Varian Eclipse TPS based on the Eclipse Scripting Application Programming Interface (ESAPI) is available at GitHub at https://github.com/44REAM/ExportFractionDose.git.

**Table 1:** Clinical and treatment characteristics of esophageal and lung cancer patients.

Clinical and Treatment Characteristics	Esophageal Cancer	Lung Cancer
	Median (Range)/n (%)	
**Age**	61 (26–93)	67 (32–87)
**Sex**		
Male	89 (0.88%)	63 (68%)
Female	12 (0.12%)	30 (32%)
**Stage**		
1	4 (4%)	31 (34%)
2	3 (3%)	58 (62%)
3	71 (70%)	3 (3%)
4	23 (23%)	1 (1%)
**Prescription dose**	50.4 (30.0–60.0)	59.4 (43.2–66)
**Prescription fraction**	1.8 (1.8–3.0)	2.0 (1.8–2.0)
**Treatment setting**		
CCRT	95 (94%)	91 (98%)
RT	6 (6%)	2 (2%)
**RT modality**		
3D conformal RT	78 (77%)	38 (41%)
IMRT/VMAT	9 (9%)	27 (29%)
Combine	14 (14%)	28 (30%)
**RT aim**		
Preoperative	47 (47%)	0 (0%)
Postoperative (adjuvant)	1 (1%)	0 (0%)
Definitive	49 (48%)	93 (100%)
Palliative	4 (4%)	0 (0%)
**RP grade**		
0	38 (38%)	77 (83%)
1	58 (57%)	
2	5 (5%)	14 (15%)
3	0 (0%)	2 (2%)
4	0 (0%)	0 (0%)

Radiation pneumonitis grading was performed by radiation oncologists based on the National Cancer Institute Common Terminology Criteria for Adverse Events version 5.0 (CTCAE v5.0). In practice, grade 0 RP was defined as negative for RP, grade 1 RP was defined as patients with symptoms or radiographic features without the need for steroids. Grade 2 RP was defined as patients requiring steroids or with symptoms that interfered with daily activities. Grade 3 RP was defined as patients requiring oxygen and steroids. Grade 4 RP was defined as patients requiring intubation. The aim of this study was to evaluate the performance of dosiomic and radiomic features for prediction of presence of any RP. However, due to unavailability of grade 1 RP data in lung cancer dataset, the positive class for esophageal cancer was defined as grade 1 or above, while for lung cancer, positive class was defined as grade 2 or above.

### Equivalent Dose in 2 Gy Fractions

Dose distributions were extracted as fractions. The dose distributions of fractions and voxels were referred to as “doses per fraction per voxel”. The equivalent dose in the 2 Gy fraction a voxel with EQD2 fractions was calculated as follows (19):


$$D_{\text{EQD}2}=\sum_{i}^{N}\frac{d_{i,k}+d_{i,k}^{2}/(\alpha /\beta )}{1+2/(\alpha /\beta )}.$$

The value of the *α*/*β* ratio in the equation was assumed to be 3 (20–26). The variable *d_i,j_* is the dose per fraction per voxel, i is the number of fractions and k is the number of voxels. The equation above was suitable for our dataset because of its compatibility with different doses per fraction per voxel. Although we used a similar prescription fraction size (1.8–3 Gy per fraction), the actual doses the patient received in different locations and with different fractions might have been different. For example, the first fraction may have been delivered by an antero-posterior beam, and the second fraction may have been delivered by 2 lateral beams, resulting in different doses per fraction for different voxels.

### Features

Resampling to 1.5 × 1.5 × 1.5 mm^3^ by b-spline algorithm was performed for all dose distributions and CT images. ROIs was resampled by nearest neighbor algorithm to match CT image. All CT images were free-breathing CT scans. The mean lung doses (MLDs), the volumes of the lungs that received doses greater than x Gy, Vx (ranging from V5 to V70 over 5 Gy steps), were used as DVH features. The Pyradiomics library in Python (27), which contains the most common feature definitions based on the Imaging Biomarker Standardization Initiative (IBSI) (28), was used to extract dosiomic and radiomic features. Dosiomic features were extracted from the resampled dose distribution. Both texture features and first-order features were then extracted from the CT images (radiomic) and dose distributions (dosiomic). The dosiomic features were extracted from lung ROIs, and the radiomics features were extracted from the lung ROIs of patients who received doses greater than x Gy for x = 10 and 20. The lung ROIs for esophageal cancer were defined as all the bilateral areas of the lungs, and that for lung cancer was defined as all the bilateral areas of the lungs minus the gross tumor volume (GTV). All ROIs were segmented by different physicians.

The dosiomic and radiomic features included in this study were based on the Pyradiomics library. However, we excluded one feature among the first-order statistics of the dosiomic features, “mean dose”, because this feature was redundant with the DVH features. All features in this study were based on 51 (17 × 3) first-order statistics features and 225 (61 × 3) texture features. The dose distributions and CT images were further processed before the calculation of dosiomic and radiomic features. The dose distribution gray-level intensity was binned to the 100 Gy level with a fixed bin size of 1 Gy. The CT image Hounsfield units (HUs) above 100 HU and below −1,000 HU were set to zero, resulting in an HU range of [−1,000 100]. Each HU value was then converted to a positive number in the range [0 1,100] and binned with a fixed bin size of 50. The texture features were based on the gray level cooccurrence matrix (GLCM) with 72 (24 × 3) features, gray level run length matrix (GLRLM) with 48 (16 × 3) features, gray level size zone matrix (GLSZM) with 48 (16 × 3) features and neighborhood gray tone difference matrix (NGTDM) with 15 (5 × 3) features. Both the DVH and dosiomic features were extracted from lung the ROIs from dose distributions with or without corrections to EQD2. All features were standardized to zero mean and unit variance. In summary, 15 DVH features, 78 dosiomic features and 156 radiomic features were extracted from each patient. The complete list of features is provided in **Supplementary Table 2**.

### Model Building

**Figure 1:**
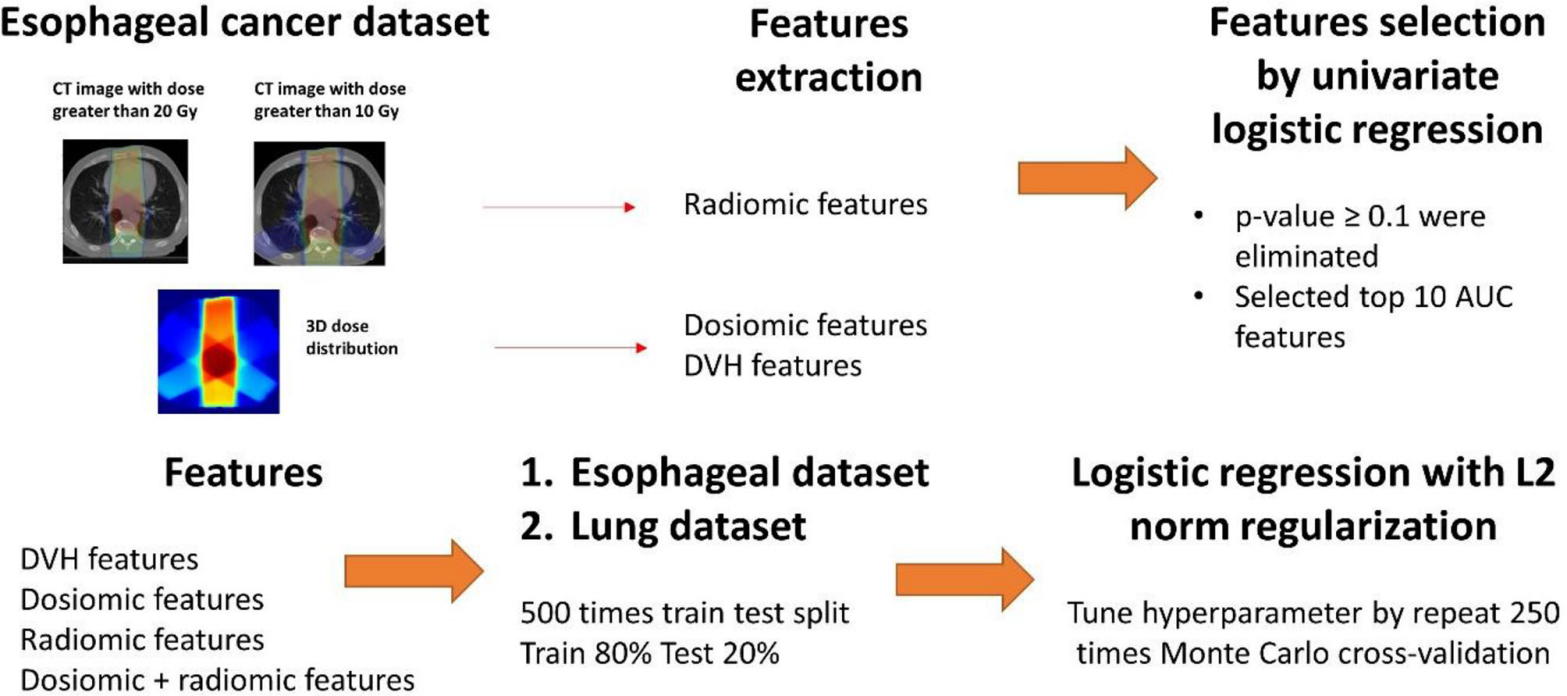
Overview of the process from inputting data to model training.

The predictive models for radiation pneumonitis were built separately for esophageal cancer patients and lung cancer patients. An overview of the process is shown in **Figure 1**.

- First, we performed feature selection *via* univariate analysis. A univariate logistic regression model was developed for all features using the entire esophageal dataset. Features that had p-values ≥0.1 were eliminated. We further trained the logistic regression model without regularization by repeat 5-fold cross-validation 50 times for the esophageal patients on the entire esophageal dataset. The top 10 features corresponding to the average area under the receiver operating characteristic curve (ROCAUC) from each feature group (DVH, dosiomic and radiomic) were selected for multivariate analysis.
- The esophageal data (500 instances) were randomly separated into a training set (80%) and test set (20%). We trained the following models: DVH (10 features), dosiomic (10 features), radiomic (10 features) and dosiomic + radiomic (20 features) models. Multivariate logistic regression with L2 norm regularization was used. The L2 norm was utilized to prevent model overfitting. The hyperparameter (regularization strength) was determined by inner cross-validation (CV) by 250 Monte Carlo CV (inner training set 80%; validation 20%) runs to maximize the average ROCAUC on the validation set using grid search. The minority class in the inner training set was oversampled by the synthetic minority oversampling technique (SMOTE) to equalize the two classes.
- Next, we trained multivariate logistic regression models using the DVH, dosiomic and radiomic features selected previously from the esophageal dataset and the lung cancer dataset. We also selected DVH features from the lung dataset (DVH_lung_) and trained the logistic regression model as described above to introduce some bias to favor the DVH features.

The model performance was evaluated by the mean ROCAUC. We also calculated the AUCs of the precision-recall curves (PRAUC) because the ROCAUC could be biased when used with imbalanced datasets (29). The mean, standard deviation (SD) and 10th–90th percentiles of the ROCAUCs and PRAUCs for the test set results of 500 models in each group were calculated. A Z-test was used to test the statistical significance of the mean AUC between each pair models. Statistical analyses were performed using the Python and SciPy packages (30). A p-value <0.05 was considered significant. For more details of the model building, we refer to **Supplementary S1**.

## Results

The selected features for the DVH, dosiomic and radiomic groups are shown in **Supplementary Table 1**. For the DVH features selected from the esophageal cancer dataset, only V45 had a p-value less than 0.1 in the univariate analysis of the lung cancer dataset. For DVH_lung_, only 3 features with p-values less than 0.1 were selected (V45, V50, and V55). The univariate analysis of lung cancer showed that 5 of 10 and 8 of 10 features had p-values less than 0.1 in the dosiomic and radiomic analyses, respectively (**Supplementary Table 1**).

**Figure 2:**
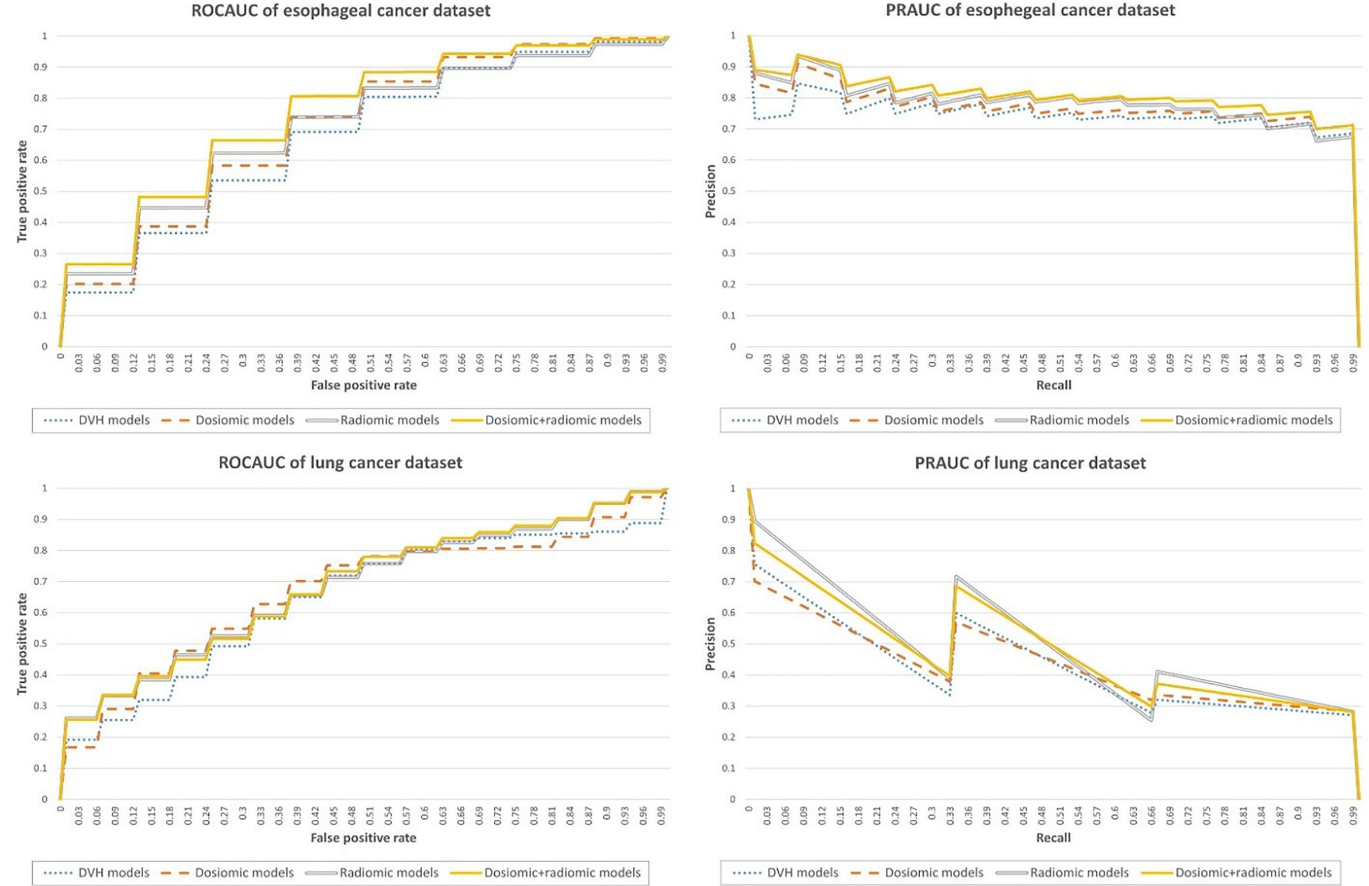
Performance metrics of the predictive models obtained on the esophageal cancer and lung cancer datasets.

The ROCAUC and PRAUC curves are shown in **Figure 2**. For the esophageal dataset, the model based on the DVH features resulted in an ROCAUC of 0.67 ± 0.11 and a PRAUC of 0.75 ± 0.10. The model based on dosiomic features resulted in an ROCAUC of 0.71 ± 0.10 and a PRAUC of 0.77 ± 0.09. The model based on radiomic features resulted in an ROCAUC of 0.71 ± 0.11 and a PRAUC of 0.79 ± 0.09. The model based on dosiomic + radiomic features resulted in an ROCAUC of 0.75 ± 0.10 and a PRAUC of 0.81 ± 0.09. The results of esophageal cancer dataset are included in **Table 2**. The ROCAUC and PRAUC of the model using dosiomic + radiomic features were significantly higher than those of the models with DVH, dosiomic and radiomic features (p-value <0.05). The AUCs of both the dosiomic model and radiomic model were also significantly higher than that of the DVH model (p-value <0.05). However, the ROCAUCs of the dosiomic and radiomic models were not significantly different (p-value = 0.62), although the PRAUC of the radiomic model was significantly higher than that of the dosiomic model (p-value <0.05).

**Table 2:** ROCAUC and PRAUC scores for the esophageal cancer dataset.

	ROCAUC	10th, 90th ROCAUC	PRAUC	10th, 90th PRAUC
DVH	0.67 ± 0.11	0.53, 0.82	0.75 ± 0.10	0.61 0.87
Dosiomic	0.71 ± 0.10	0.58, 0.84	0.77 ± 0.09	0.65 0.89
Radiomic	0.71 ± 0.11	0.57, 0.85	0.79 ± 0.09	0.66 0.90
Dosiomic + Radiomic	0.75 ± 0.10	0.63, 0.88	0.81 ± 0.09	0.68, 0.92

For the lung dataset, the model based on DVH features resulted in an ROCAUC of 0.64 ± 0.18 and a PRAUC of 0.37 ± 0.20. The model based on dosiomic features resulted in an ROCAUC of 0.67 ± 0.17 and a PRAUC of 0.37 ± 0.20. The model based on radiomic features resulted in an ROCAUC of 0.67 ± 0.16 and a PRAUC of 0.45 ± 0.23. The model based on dosiomic + radiomic features resulted in an ROCAUC of 0.68 ± 0.16 and a PRAUC of 0.44 ± 0.22. The results of lung cancer dataset were included in **Table 3**. The ROCAUCs of the dosiomic, radiomic and dosiomic + radiomic models was significantly higher than that of the DVH model. However, only the PRAUCs of the radiomic and dosiomic + radiomic models were significantly higher than that of the DVH model, and the PRAUCs of the dosiomic and DVH models were not significantly different (p-value = 0.61).

**Table 3:** ROCAUC and PRAUC scores for the lung cancer dataset.

	ROCAUC	10th, 90th ROCAUC	PRAUC	10th, 90th PRAUC
DVH_lung_	0.64 ± 0.18	0.42, 0.88	0.38 ± 0.20	0.13, 0.66
DVH	0.64 ± 0.18	0.42, 0.88	0.37 ± 0.20	0.13, 0.66
Dosiomic	0.67 ± 0.17	0.44, 0.90	0.37 ± 0.20	0.15, 0.68
Radiomic	0.67 ± 0.16	0.46, 0.88	0.45 ± 0.23	0.14, 0.75
Dosiomic + Radiomic	0.68 ± 0.16	0.46, 0.90	0.44 ± 0.22	0.15, 0.75

We also provided the results when selecting the features within CV loop in **Supplementary Tables 3–5**. The model building method was included in **Supplementary S2**. For esophageal dataset, the model based on the DVH features resulted in an ROCAUC of 0.67 ± 0.10 and a PRAUC of 0.74 ± 0.09. The model based on dosiomic features resulted in an ROCAUC of 0.70 ± 0.10 and a PRAUC of 0.77 ± 0.09. The model based on radiomic features resulted in an ROCAUC of 0.63 ± 0.11 and a PRAUC of 0.72 ± 0.09. The model based on dosiomic + radiomic features resulted in an ROCAUC of 0.70 ± 0.11 and a PRAUC of 0.78 ± 0.10. For lung cancer dataset, the model based on the DVH features resulted in an ROCAUC of 0.61 ± 0.17 and a PRAUC of 0.26 ± 0.16. The model based on dosiomic features resulted in an ROCAUC of 0.67 ± 0.18 and a PRAUC of 0.38 ± 0.21. The model based on radiomic features resulted in an ROCAUC of 0.66 ± 0.17 and a PRAUC of 0.46 ± 0.24. The model based on dosiomic + radiomic features resulted in an ROCAUC of 0.70 ± 0.17 and a PRAUC of 0.43 ± 0.22.

## Discussion

Our results showed that the dosiomic and radiomic models achieved higher AUCs than the DVH-based models on the esophageal cancer dataset. The results from our studies, obtained on an esophageal cancer dataset, were different from those of previous studies on the use of quantitative CT image features for esophageal cancer to predict RP grade ≥2, which found that SUV95 was a predictive feature but that CT images were bad predictors (12). However, we investigated more CT radiomic features than a previous study by Castillo et al. with respect to esophageal cancer. Furthermore, in our population, PET might not have been available for all patients due to restrictions in Thailand. Another study on esophageal cancer data also found that CT image-based delta-radiomics improved discriminative ability of patient developing grade ≥ 3RP within 3 months (31). Delta-radiomics was the technique that analyzes the radiomics features at different time. It was show that delta-radiomics features were robust than simple radiomics features (32). Delta-radiomics also have the advantage of more data over radiomics features. An advantage of using only pre-treatment data is that it might allow clinician to revise the treatment plan before initiating treatment, therefore preventing radiation pneumonitis.

In the lung dataset, only the radiomic and dosiomic + radiomic models achieved better performance than the DVH-based model. Although the ROCAUC of the dosiomic model was better than that of the DVH-based model, the PRAUC of the dosiomic-based model was not different from that of the DVH-based model. This demonstrated that dosiomics and radiomics could improve the performance of predictive models for RP, as observed in previous studies (6, 33, 34). Furthermore, knowledge of radiomic and dosiomic features might be transferable from one dataset to another dataset with performance that is equivalent to or better than that of standard DVH features.

The DVH features selected using the lung cancer dataset (DVH_lung_) were expected to differ from the DVH features selected from the esophageal cancer dataset, and the model using DVH_lung_ was expected to have better performance than the DVH features selected from the esophageal cancer dataset. From the results, DVH_lung_ was different from DVH, as expected, but the performance of DVH was not different from that of DVH_lung_. Nonetheless, radiomics and dosiomics still performed slightly better regarding the prediction of RP than DVH_lung_.

Previous studies on the use of radiomics and dosiomics for radiation pneumonitis prediction reported a variety of “most important” features. Among CT radiomic studies with respect to RP, Hirose et al. were the first to investigate a predictive model using only pretreatment CT radiomics for predicting RP grade ≥2 in lung cancer after stereotactic body radiotherapy (SBRT) (6). One of the most selected features was “correlation” from the GLCM. Nevertheless, a radiomic study by Krafft et al. (7) did not find any features that were common with those of Hirose et al. It was difficult to compare the two studies since the treatment modalities, extracted features and methods employed to build the models were not the same. The results of dosiomic studies relative to RP were also difficult to compare due to differences in the extracted features. For example, Liang et al. found “contrast” from the GLCM and “low grey level run emphasis” from the GLRLM as the most predictive features of RP ≥2 in lung cancer patients treated with volumetric modulated arc therapy (VMAT) (34), while the study of dosiomics in lung cancer patients treated with VMAT by Bourbonne et al. investigated acute and late lung toxicity separately, which was different approach from that of Liang et al. (35). Adachi et al. made a study of dosiomics that utilized different modalities (SBRTs) and different techniques for feature extraction (33).

A systemic review of PET/CT texture features also found that many texture features have been reported even though the datasets used were similar in terms of cancer types and modalities (13). The study in which the dataset and features were the most similar to those in our study was the work of Liang et al., although the patients were different, and there was no work done regarding CT radiomics (34). One drawback of their study was that the result was not validated on a test set. However, we separated a test dataset for the evaluation of our model. From the obtained results, our selected dosiomic features were different from those of their study. The differences in the selected features might be derived from the difference between the training sets, since we trained the model on esophageal cancer patients, as opposed to lung cancer patients.

Some studies have reported that the results from radiomic features can be biased due to false positives, and an external dataset is required to confirm the predictability of models (13, 36). Our study did not use an external dataset to validate the performance of the predictive model but to validate the radiomic and dosiomic features instead. To avoid biases in the radiomic and dosiomic features, we incorporated lung cancer patients receiving RT as an external dataset with an end point of predicted RP grade ≥2. The aim of incorporating an external dataset was to ensure the superiority of the predictive abilities of dosiomic and radiomic features over that of DVH features in the same organ. The results obtained on an external dataset indicated that dosiomic and radiomic feature performed equally or superior to DVH-based features in the same organ, even with different primary cancers.

There were several limitations in our study. First, our study was based on retrospective data, which might have resulted in false positives (36). Nevertheless, we tried to overcome this limitation by introducing lung cancer patients as an external dataset. Another limitation was that, from a biological standpoint, grade 1 RP and grade 2 RP are different. This is due to unavailability of grade 1 RP data in lung cancer. Grade 1 RP is viewed as local damage from the criterion of CT image changes. This might be a drawback of the study regarding grade 1 RP, which could cause the results to be inapplicable to grade 2 RP. However, grade 1 RP can also be viewed as whole organ damage if it is classified as grade 1 by symptom criteria, which would be biologically similar to grade 2 RP. The input spaces in the lung cancer and esophageal cancer datasets were also different. The input space in the lung cancer dataset had smaller ROIs than that in the esophageal cancer dataset, and the doses administered to the lungs of lung cancer patients were also higher than the doses administered to the lungs of esophageal cancer patients. Another difference was that the risk factors, such as the locations of the primary tumors, were not the same. Despite all the differences described above, we found that the dosiomic and radiomic feature models could achieve performance that was equal or superior to that of the DVH model.

The method of features selection in esophageal cancer dataset might cause overfitting problem for esophageal cancer results since the features selection process was carry out of the CV loop but not be for lung cancer dataset. Thus, we also provided the results of models using feature selection within the CV loop which eliminated the problem of overfitting in **Supplementary S2**. In summary, only the performance of the model that included radiomic decrease less than DVH model in esophageal cancer dataset (**Supplementary Table 3**), while in lung cancer dataset, the performance of radiomic and dosiomic model still greater than DVH model (**Supplementary Table 4**). The featured select in both methods were similar (**Supplementary Table 5**). The purpose was to test the transferability of dosiomic and radiomic features to lung cancer dataset which the process does not cause overfitting in the lung cancer results.

## Conclusion

In conclusion, studies on the dosiomic and radiomic feature of RP are in the early stage. Our study found that dosiomic and radiomic models could enhance the performance of RP prediction models for esophageal and lung cancer patients treated with RT. Further prospective studies are required to validate the effectiveness of dosiomic and radiomic features.

## Data Availability Statement

The data analyzed in this study is subject to the following licenses/restrictions: No. Requests to access these datasets should be directed to yodchanan.won@mahidol.ac.th.

## Ethics Statement

The studies involving human participants were reviewed and approved by the Human research ethics committee, Faculty of Medicine Ramathibodi Hospital, Mahidol University (IRB MURA2021/283). Written informed consent for participation was not required for this study in accordance with the national legislation and the institutional requirements.

## Author Contributions

CP, NS, NT and CJ contributed to the acquisition of the data. NS and PP contributed to clinical criteria. CP contributed to processing the data, features extraction, machine learning, statistical analysis and drafted the manuscript. SK, PP, CJ and YW were senior author supervising the project.

## Funding

This study is supported in part by the National Higher Education Science Research and Innovation Policy Council, PMU B (B05F640079).

## Conflict of Interest

The authors declare that the research was conducted in the absence of any commercial or financial relationships that could be construed as a potential conflict of interest.

## Publisher’s Note

All claims expressed in this article are solely those of the authors and do not necessarily represent those of their affiliated organizations, or those of the publisher, the editors and the reviewers. Any product that may be evaluated in this article, or claim that may be made by its manufacturer, is not guaranteed or endorsed by the publisher.
