# Supplementary material for: Radiomic and Dosiomic Features for the Prediction of Radiation Pneumonitis Across Esophageal Cancer and Lung Cancer
Source: Front Oncol. 2022 Feb 16;12:768152. doi: 10.3389/fonc.2022.768152 (PMC8889567; doi:10.3389/fonc.2022.768152)
Supplement: Supplementary file 1 [file DataSheet_1.docx]

**S1 Model building**

For binary classification objective function of logistic regression model with L2 norm regularization can be written as below:

$$L=x=\frac{1}{2}\left| \left| w \right| \right|_{2}+C\sum_{i} log\left( exp\left( -y_{i}\left( X_{i}^{T}w+b \right) \right)+1 \right)$$

Where w and b are model coefficients, $X_{i}$ and $y_{i}$ is input features and input class for patient $i$ respectively. Parameter C is hyperparameter of L2 norm regularization. Strength of regularization is proportional to 1/C. The hyperparameter of logistic regression with L2 norm regularization model was tune by grid search for C $\in$ [0.001, 0.01, 0.1, 1] by selecting C that maximize mean AUC of 250 round of Monte Carlo cross-validation

Models were train in Python using Sklearn library. Other hyperparameter in model are optimizer, which call “solver” in Sklearn, was set to “liblinear” and maximum iteration, which call “max_iter” was set to 10000, the remaining hyperparameter are set to default.

**Supplementary table 1: p-value of univariate logistic regression.**

| **features** | **p-value (esophageal cancer)** | **p-value (lung cancer)** |
| --- | --- | --- |
| **DVH_lung_** | | |
| V50 |  | 0.022 |
| V45 |  | 0.052 |
| V55 |  | 0.040 |
| **DVH** | | |
| MLD | 0.002 | 0.120 |
| V40 | 0.003 | 0.142 |
| V35 | 0.003 | 0.152 |
| V45 | 0.013 | 0.052 |
| V20 | 0.007 | 0.228 |
| V30 | 0.009 | 0.138 |
| V25 | 0.009 | 0.161 |
| V10 | 0.013 | 0.724 |
| V5 | 0.019 | 0.904 |
| V15 | 0.010 | 0.427 |
| **Dosiomic** | | |
| NGTDM Busyness | <0.001 | 0.106 |
| GLCM SumAverage | 0.002 | 0.050 |
| GLCM JoinAverage | 0.002 | 0.050 |
| 90 percentiles | 0.002 | 0.054 |
| GLCM Autocorrelation | 0.002 | 0.022 |
| NTGDM Contrast | 0.002 | 0.166 |
| RootMeanSquare | 0.001 | 0.031 |
| GLCM DifferenceAverage | 0.003 | 0.248 |
| GLSZM LowGrayLevelZoneEmphasis | 0.003 | 0.763 |
| GLSZM SmallAreaLowGrayLevelEmphasis | 0.006 | 0.817 |
| **Radiomic** | | |
| TotalEnergy_V20 | 0.002 | 0.010 |
| Energy_V20 | 0.002 | 0.010 |
| Energy_V10 | 0.010 | 0.013 |
| TotalEnergy_V10 | 0.010 | 0.013 |
| NGTDM Stength_V20 | 0.013 | 0.402 |
| GLCM SumSquares_V20 | 0.028 | 0.003 |
| GLCM ClusterPorminence_V20 | 0.087 | 0.020 |
| GLRLM RunLengthNonUniformity_V20 | 0.006 | 0.640 |
| GLSZM SmallAreaHighGrayLevelEmphasis_V20 | 0.018 | 0.082 |
| GLCM ClusterTendency_V20 | 0.028 | 0.003 |

**Supplementary table 2: all features use in this study.**

|  | Features |
| --- | --- |
| DVH features (15 features) | Mean lung dose (MLD), V5 – V70 |
| First Order (17 features) | **Energy, Total Energy, Entropy, Minimum, 10th percentile, 90th percentile, Maximum, Median, Interquartile Range, Range, Mean Absolute Deviation (MAD), Robust Mean Absolute Deviation (rMAD), Root Mean Squared (RMS), Skewness, Kurtosis, Variance, Uniformity** |
| Gray Level Co-occurrence Matrix (GLCM) (24 features) | **Autocorrelation, Joint Average, Cluster Prominence, Cluster Shade, Cluster Tendency, Contrast, Correlation, Difference Average, Difference Entropy, Difference Variance, Joint Energy, Joint Entropy, Informational Measure of Correlation (IMC), Informational Measure of Correlation (IMC) 2, Inverse Difference Moment (IDM), Maximal Correlation Coefficient (MCC), Inverse Difference Moment Normalized (IDMN), Inverse Difference (ID), Inverse Difference Normalized (IDN), Inverse Variance, Maximum Probability, Sum Average, Sum Entropy, Sum of Squares** |
| Gray Level Run Length Matrix (GLRLM) (16 features) | **Short Run Emphasis (SRE), Long Run Emphasis (LRE), Gray Level Non-Uniformity (GLN), Gray Level Non-Uniformity Normalized (GLNN), Run Length Non-Uniformity (RLN), Run Length Non-Uniformity Normalized (RLNN), Run Percentage (RP), Gray Level Variance (GLV), Run Variance (RV), Run Entropy (RE), Low Gray Level Run Emphasis (LGLRE), High Gray Level Run Emphasis (HGLRE), Short Run Low Gray Level Emphasis (SRLGLE), Short Run High Gray Level Emphasis (SRHGLE), Long Run Low Gray Level Emphasis (LRLGLE), Long Run High Gray Level Emphasis (LRHGLE)** |
| Gray Level Size Zone Matrix (GLSZM) (16 features) | **Small Area Emphasis (SAE), Large Area Emphasis (LAE), Gray Level Non-Uniformity (GLN), Gray Level Non-Uniformity Normalized (GLNN), Size-Zone Non-Uniformity (SZN), Size-Zone Non-Uniformity Normalized (SZNN), Zone Percentage (ZP), Gray Level Variance (GLV), Zone Variance (ZV), Zone Entropy (ZE), Low Gray Level Zone Emphasis (LGLZE), High Gray Level Zone Emphasis (HGLZE), Small Area Low Gray Level Emphasis (SALGLE), Small Area High Gray Level Emphasis (SAHGLE), Large Area Low Gray Level Emphasis (LALGLE), Large Area High Gray Level Emphasis (LAHGLE)** |
| Neighbouring Gray Tone Difference Matrix (NGTDM) (5 features) | Coarseness, Contrast, Busyness, Complexity, Strength |

Note that each of features in the table S3 except DVH features are multiply by 3. This is because one feature come from 1 dosiomic and 2 radiomic features. For “2” radiomic features, “2” come from the extraction the features from lung ROIs that receive dose greater than 10 Gy and 20 Gy. For example, if we want to extract “**Energy**” from “first order” features, we extract one from dose distribution in lung ROIs and two from CT image with lung ROIs that greater than 10 Gy and 20 Gy. The features extract from CT image were written in the form of “{featurename}_Vx” for x is 10 or 20.

**S2 Others model**

The model building process was similar to the original paper except:

1. Firstly, for the entire dataset, we removed features with correlation coefficient greater than 0.9. For two correlated features, the feature which had a greater number of correlation with other features were removed. The features selection process was carried out within CV loop for esophageal cancer dataset. For lung dataset, we use the top 10 most selected features with frequency of being selected greater than 60% in the esophageal cancer dataset.
2. We did train-test split for 200 times as opposed to 500 times in the original paper for outer CV loop. For inner CV loop, we the split was done for 100 times as opposed to 250 times in the original paper. The model performance results were shown in Supplementary Table 3 and 4. Features which have been selected more than 60% of frequency were shown in Supplementary table 5.

The model performance results were showed in Supplementary table 3 and 4. The features which have been selected more than 60% of frequency were showed in Supplementary table 5.

**Supplementary table 3: ROCAUC and PRAUC scores for the esophageal cancer dataset using features selection within CV loop**

|  | ROCAUC | 10^th^, 90^th^ ROCAUC | PRAUC | 10^th^, 90^th^ PRAUC |
| --- | --- | --- | --- | --- |
| DVH | 0.67**±**0.10 | 0.54, 0.80 | 0.74**±**0.09 | 0.62 0.86 |
| Dosiomic | 0.70**±**0.10 | 0.57, 0.84 | 0.77**±**0.09 | 0.65 0.88 |
| Radiomic | 0.63**±**0.11 | 0.49, 0.77 | 0.72**±**0.09 | 0.60 0.84 |
| Dosiomic + Radiomic | 0.70**±**0.11 | 0.59, 0.84 | 0.78**±**0.10 | 0.65, 0.90 |

**Supplementary table 4: ROCAUC and PRAUC scores for the lung cancer dataset using features selection within CV loop**

|  | ROCAUC | 10^th^, 90^th^ ROCAUC | PRAUC | 10^th^, 90^th^ PRAUC |
| --- | --- | --- | --- | --- |
| DVH | 0.61±0.17 | 0.35, 0.83 | 0.26±0.16 | 0.13, 0.48 |
| Dosiomic | 0.67±0.18 | 0.42, 0.90 | 0.38±0.21 | 0.15, 0.72 |
| Radiomic | 0.66±0.17 | 0.44, 0.88 | 0.46±0.24 | 0.13, 0.76 |
| Dosiomic + Radiomic | 0.70±0.17 | 0.48, 0.92 | 0.43±0.22 | 0.15, 0.73 |

**Supplementary table 5: The features which have been selected more than 60% of frequency**

| **DVH** |
| --- |
| MLD |
| V40 |
| V45 |
| V20 |
| V10 |
| V5 |
| V15 |
| **Dosiomic** |
| NGTDM Busyness |
| GLCM SumAverage |
| 90 percentiles |
| NTGDM Contrast |
| GLSZM LowGrayLevelZoneEmphasis |
| **GLCM_InverseDifferenceNormalized (Idn)** |
| **Radiomic** |
| TotalEnergy_V20 |
| TotalEnergy_V10 |
| NGTDM Stength_V20 |
| GLRLM_GrayLevelVariance_V20 |
